# MV-CVIB: **a** microbiome-based multi-view convolutional variational information bottleneck for predicting metastatic colorectal cancer

**DOI:** 10.3389/fmicb.2023.1238199

**Published:** 2023-08-22

**Authors:** Zhen Cui, Yan Wu, Qin-Hu Zhang, Si-Guo Wang, Ying He, De-Shuang Huang

**Affiliations:** ^1^Institute of Machine Learning and Systems Biology, College of Electronics and Information Engineering, Tongji University, Shanghai, China; ^2^College of Electronics and Information Engineering, Tongji University, Shanghai, China; ^3^EIT Institute for Advanced Study, Ningbo, Zhejiang, China

**Keywords:** microbiome, multi-view, information bottleneck, metastatic colorectal cancer, risk assessment

## Abstract

**Introduction:**

Imbalances in gut microbes have been implied in many human diseases, including colorectal cancer (CRC), inflammatory bowel disease, type 2 diabetes, obesity, autism, and Alzheimer's disease. Compared with other human diseases, CRC is a gastrointestinal malignancy with high mortality and a high probability of metastasis. However, current studies mainly focus on the prediction of colorectal cancer while neglecting the more serious malignancy of metastatic colorectal cancer (mCRC). In addition, high dimensionality and small samples lead to the complexity of gut microbial data, which increases the difficulty of traditional machine learning models.

**Methods:**

To address these challenges, we collected and processed 16S rRNA data and calculated abundance data from patients with non-metastatic colorectal cancer (non-mCRC) and mCRC. Different from the traditional health-disease classification strategy, we adopted a novel disease-disease classification strategy and proposed a microbiome-based multi-view convolutional variational information bottleneck (MV-CVIB).

**Results:**

The experimental results show that MV-CVIB can effectively predict mCRC. This model can achieve AUC values above 0.9 compared to other state-of-the-art models. Not only that, MV-CVIB also achieved satisfactory predictive performance on multiple published CRC gut microbiome datasets.

**Discussion:**

Finally, multiple gut microbiota analyses were used to elucidate communities and differences between mCRC and non-mCRC, and the metastatic properties of CRC were assessed by patient age and microbiota expression.

## 1. Introduction

The human intestine is one of the most important organs in the digestive system, which maintains the normal life activities of the human body through metabolism (Cho and Blaser, 2012). Microbes in the gut derive energy from the food we eat and release metabolites and hormones to regulate physical health. As our microbial research continues to deepen, more and more investigations show that the chemical signals released by human gut microbes play a key role in human health and disease (Gilbert et al., 2018). From the perspective of human health, the intestinal flora in the body contributes to the construction of the immune system and participates in and regulates the physiological processes of various cells (De Sordi et al., 2019). More importantly, a variety of complex diseases have been confirmed to be related to certain intestinal flora, including inflammatory bowel disease, type 2 diabetes, Alzheimer's disease, HIV, autism, obesity, and cardiovascular and cerebrovascular diseases (Schmidt et al., 2018; Shkoporov et al., 2019). Some malignancies, such as colorectal cancer (CRC), have also been shown to be associated with gut microbes (Chen et al., 2022; Wani et al., 2022). CRC is the third leading cause of cancer deaths, and ~20% of patients develop metastases, known as metastatic colorectal cancer (mCRC). It mainly includes colon cancer liver metastasis, multiple lymph node metastasis, hematogenous metastasis, and implantation metastasis (Enquist et al., 2014). All of this emerging evidence confirms that the gut microbiome can be a potential predictor of a variety of diseases and cancers (Zou et al., 2017).

With the advent of the genome era, the development of high-throughput sequencing technology has provided a new technical platform for the study of microbial community structure (Zhou et al., 2015). In particular, 16S rRNA gene sequencing has become an important means to study the composition and distribution of gut microbial communities (Langille et al., 2013). It fully shows the diversity of human gut flora and reveals potential factors for disease aggravation. Although much evidence suggests that the gut microbiome can be used to predict colorectal cancer, few investigations have used microbial data to identify mCRC. Therefore, effectively extracting key features of the microbiota from gut microbial data faces a series of challenges (Cammarota et al., 2020; Wang and Zou, 2023). Since disease samples are small and more difficult to obtain than healthy samples, a large number of studies use healthy-disease groups rather than disease-disease groups. A small number of samples and many features can lead to the curse of dimensionality, that is, features are highly sparse, such as strain-level informative data containing hundreds of thousands of genetic markers (Somorjai et al., 2003; Akay and Hess, 2019). However, it is almost difficult for traditional machine learning models to mine valuable information from such small sample data. Second, although gene signatures provide more information than microbial abundance data, more feature information also requires huge computational resources, which may lead to overfitting and greatly increase the time cost (Yang et al., 2021).

Considering the metastatic characteristics of CRC, non-metastatic colorectal cancer (non-mCRC) patients are more worried about mCRC with higher mortality (Reyes et al., 2019; Rumpold et al., 2020). Existing microbiome-based CRC prediction methods mainly use species relative abundance or strain-level marker profiles or a combination of the two. With the development of deep learning, it has become feasible to use deep learning to predict CRC from gut microbiome data (Marcos-Zambrano et al., 2021; Salim et al., 2023). The MicroPheno method is based on 16S rRNA sequence data, subsamples it, and computes the k-mer representation of the sequence, and the final k-mer is used to complete disease prediction (Asgari et al., 2018). Oh and Zhang (2020) proposed dimensionality reduction of microbiome abundance data or gene signature profiles with multiple different autoencoders, and then classical machine learning methods were used to complete disease classification. Reiman et al. (2020) took a microbial phylogenetic tree matrix as input and used a convolutional neural network (CNN) for disease prediction. Wirbel et al. (2021) developed SIAMCAT, a multifunctional R toolbox for machine learning-based comparative metagenomics. The toolbox contains a variety of feature matrices such as genes, pathways, and microbial taxa to statistically infer host disease phenotype associations. Grazioli et al. proposed multimodal variational information bottleneck (MVIB), a multimodal representation that can input species relative abundance, strain-level marker profiles, and metabolomic data and learn meaningful joint codes through information bottleneck theory (Grazioli et al., 2022). This study used multiple published microbiome disease phenotype datasets including CRC and achieved excellent predictive performance. However, the relative independence among relative abundances, strain-level marker profiles, and metabolomic data contains rich cross-modal information in addition to the modal information of the microbiome, which may lead to model uncertainty (Holzinger et al., 2022).

**Figure 1:**
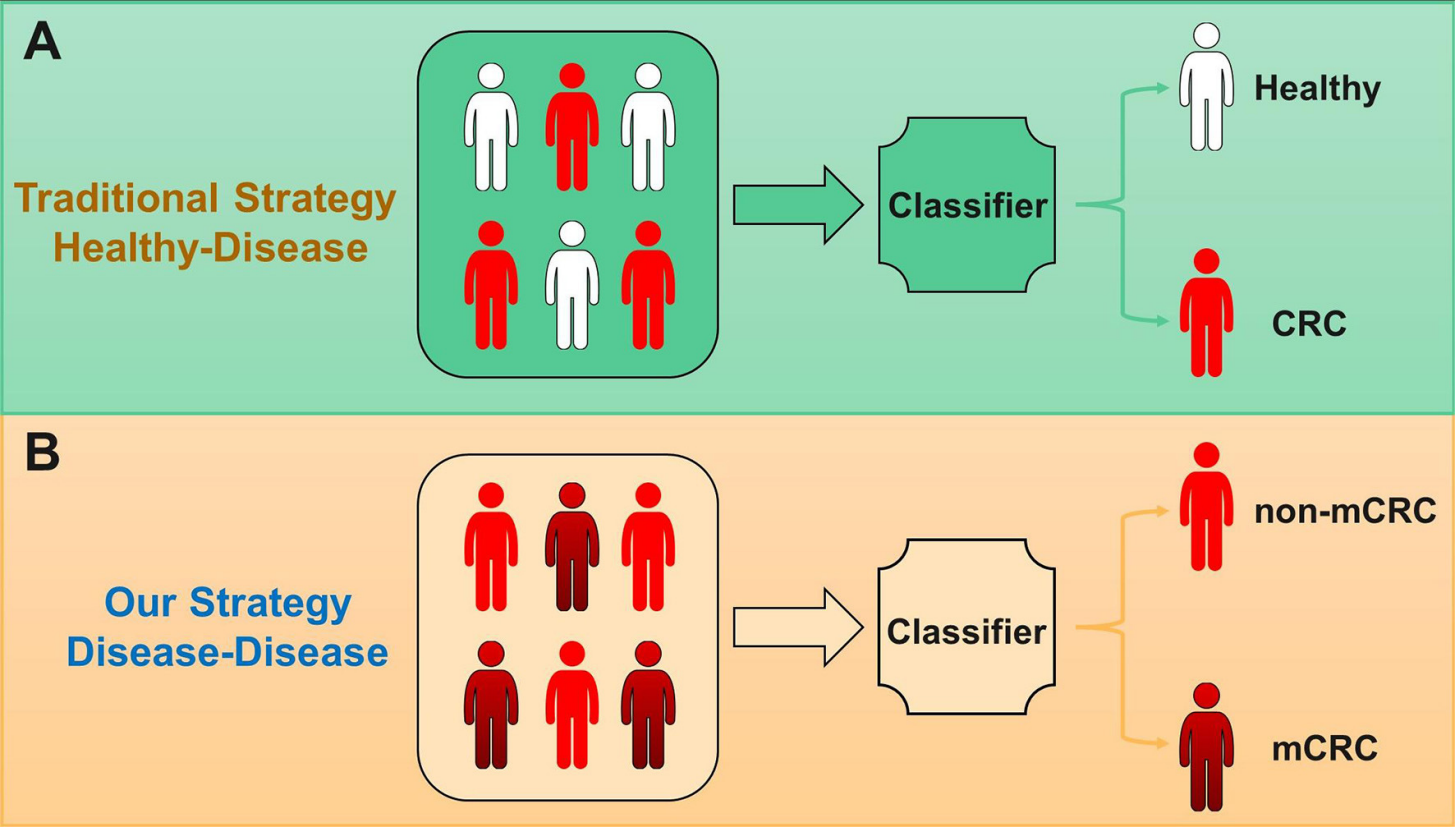
**(A)** Traditional classification strategy for microbiome-based disease prediction is health to disease. **(B)** Our strategy for microbiome-based disease prediction is disease to disease, focusing on predicting malignancy from disease, such as the prediction from non-metastatic colorectal cancer (non-mCRC) to metastatic colorectal cancer (mCRC).

Compared with the traditional health-disease classification strategy, we adopt a new disease-disease classification strategy, which identifies more severe diseases among sick patients. Disease-disease sample features are often more difficult to distinguish than healthy-disease sample features, which is also a challenge for predictive models. Figure 1 shows the specific strategy process.

In this study, we propose a multi-view convolutional variational information bottleneck (MV-CVIB) model to specifically address the prediction problem of mCRC. The variational information bottleneck (VIB) can extract all the judgmental information that is helpful for disease prediction while filtering out redundant information (Alemi et al., 2016). For deep neural networks, forgetting details enables the model to form general concepts and improves generalization performance. The Qiime2 tool was used to process and obtain the final relative abundance data (Hall and Beiko, 2018). We calculated the Euclidean distance between each sample based on the relative abundance of the microbiome and took the samples with the closest Euclidean distance as neighbors. Therefore, the nearest neighbor information between each sample can be regarded as a new view. MV-CVIB expands the microbiome input data structure to the maximum capacity while also being insensitive to outliers in the data. Not only that, to test the generalization ability of MV-CVIB, we also performed various experiments on multiple published control-CRC datasets.

The contributions of this study are as follows:

1) Current studies mainly focus on the prediction of CRC while neglecting the more severe mCRC. We are the first to apply deep learning to the microbiome-based mCRC prediction problem and achieve excellent prediction results.
2) Compared with the traditional health-disease classification strategy, we adopt a new disease-disease classification strategy, which identifies more severe diseases among sick patients. Identifying more complex diseases from diseases is more conducive to mining the underlying nature of disease exacerbations.
3) We compute the nearest neighbor information for the relative abundance data and feed it together as a view into the VIB with convolution and pooling modules. The VIB can extract all the judgmental information that is helpful for disease prediction while filtering out redundant information. Since we introduce convolution and pooling operations into the model, the data in the input stream become smoother, which increases the robustness and generalization ability of the model and avoids overfitting.

## 2. Materials and methods

### 2.1. Datasets

**Figure 2:**
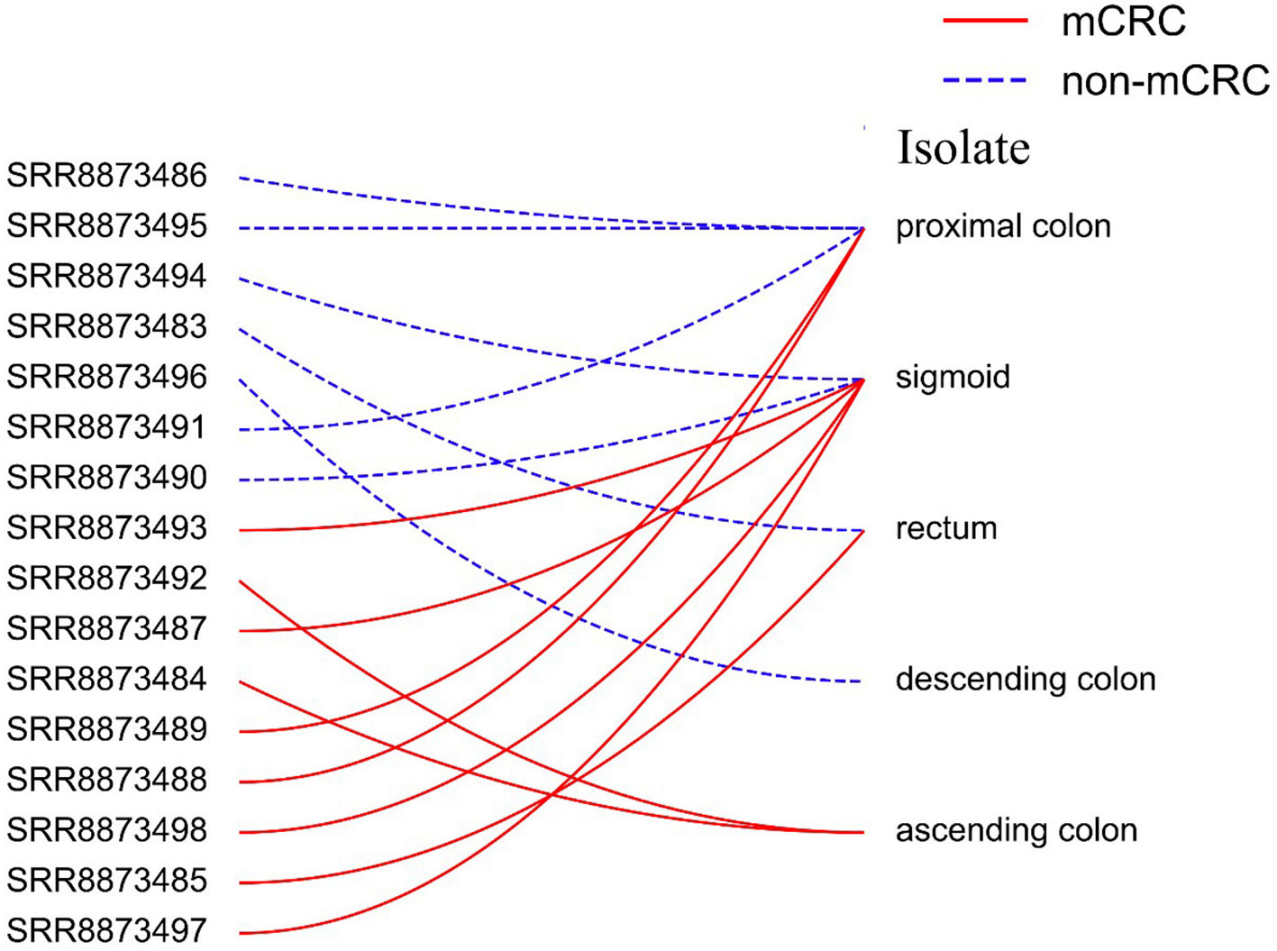
Each sample is isolated differently from the gut.

To evaluate and analyze predictive models, we collected 16S rRNA data from the gut microbiota of patients with metastatic colorectal cancer (mCRC, *n* = 9) and non-metastatic colorectal cancer (non-mCRC, *n* = 7) from the National Center for Biotechnology Information (NCBI) (Coordinators, 2015). The original data come from the People's Hospital of Wuhan University, and the data type is raw sequence read. Raw sequence data can be accessed through the NCBI Sequence Read Archive (SRA) database (https://www.ncbi.nlm.nih.gov/bioproject/?term=PRJNA531761). Table 1 shows the specific information of all samples. Figure 2 shows that each sample is isolated in the gut. The pre-processing of this dataset will be described in detail in the next section.

**Table 1:** Specific information for non-mCRC patients and mCRC patients.

**Sample ID**	**Disease status**	**Age**	**Gender**
SRR8873486	Non-mCRC	68	Male
SRR8873495	Non-mCRC	74	Male
SRR8873494	Non-mCRC	68	Male
SRR8873483	Non-mCRC	65	Female
SRR8873496	Non-mCRC	66	Female
SRR8873491	Non-mCRC	38	Male
SRR8873490	Non-mCRC	54	Female
SRR8873493	mCRC	44	Female
SRR8873492	mCRC	70	Male
SRR8873487	mCRC	82	Female
SRR8873484	mCRC	85	Male
SRR8873489	mCRC	56	Female
SRR8873488	mCRC	73	Male
SRR8873498	mCRC	32	Male
SRR8873485	mCRC	61	Female
SRR8873497	mCRC	68	Male

In addition, we also collected three control-CRC datasets from published studies to evaluate the generalization ability of the model, specifically including colorectal (Pasolli et al., 2016), colorectal-EMBL, and early-colorectal-EMBL (Zeller et al., 2014). Only the CRC group and the healthy group were included in these datasets and had unique true labels, diseased or healthy. Therefore, this study does not perform predictions on the future health of the samples. Table 2 shows the specific information of the three public datasets.

**Table 2:** Three CRC datasets from published studies.

**Datasets**	**Total samples**	**Control sample**	**Disease samples**
Colorectal (CRC1)	121	73	48
Colorectal-EMBL (CRC2)	199	103	96
Early-colorectal-EMBL (CRC3)	96	52	44

### 2.2. Pre-processing

First, we converted sra files on NCBI to fastq.gz files using fastq-dump version 2.8.0 in SRA Toolkit and then converted it to multiple fastq files with forward and reverse. The next step is to import these data into Qiime2 and review the data quality. Next, we denoise the data using Deblur with default parameters. The specific role is to filter out noisy sequences, remove chimeric sequences, accidental sequences (sequences that occur only once), and de-redundant these sequences. The purpose is to obtain the signature table and reference sequence.

Finally, we used Qiime2 to analyze the composition of microbial communities from the denoised data. Among them, the feature table represents the relative abundance of species and serves as the input feature vector of the proposed model.

### 2.3. Multiple types of dimensionality reduction analysis

**Figure 3:**
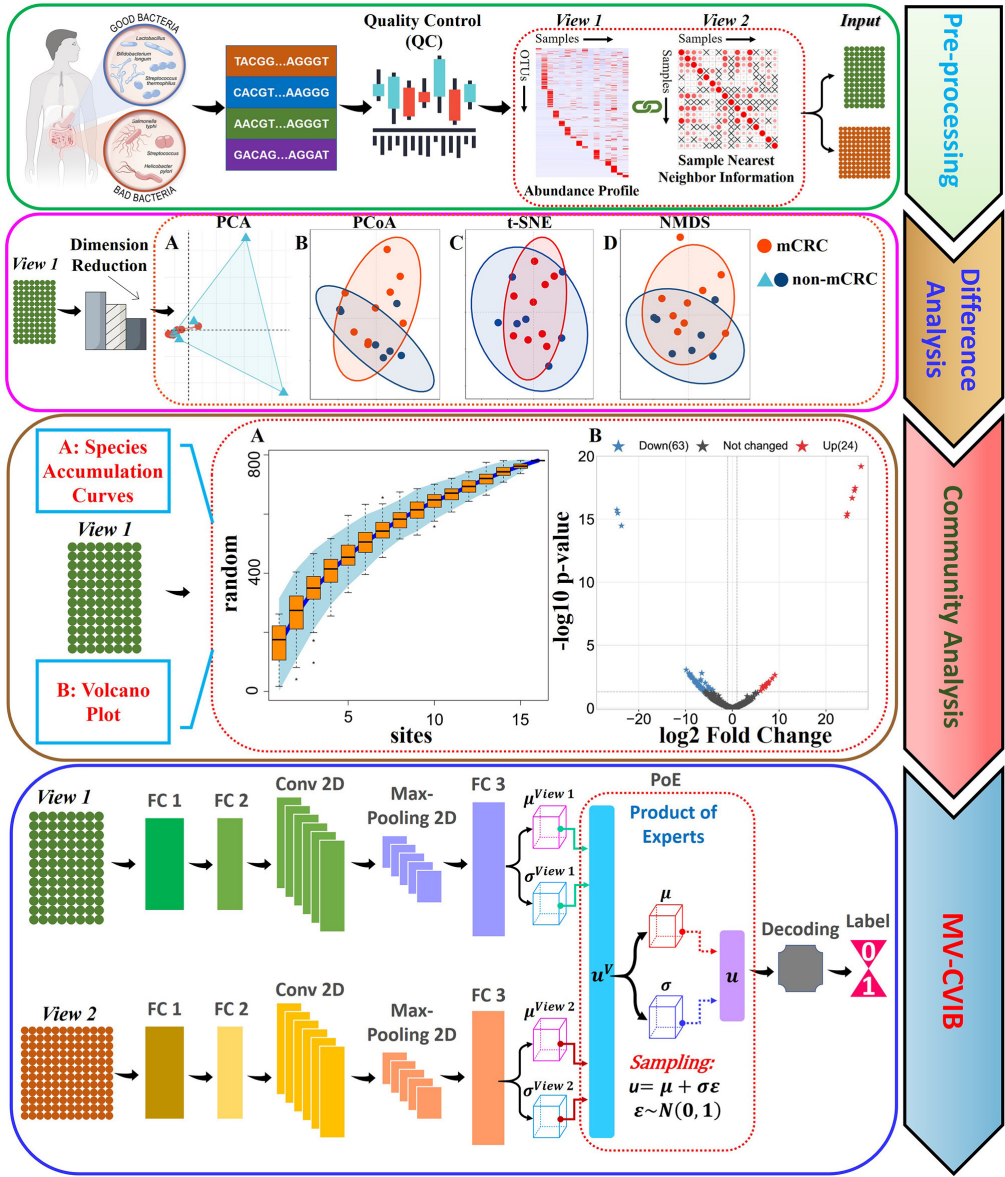
Work flowchart. Pre-processing: We collected the sequence information of the samples from NCBI and obtained the species abundance data through a series of quality control and filtering methods, and at the same time, we calculated the nearest neighbor information of the samples. Variance Analysis: We map abundance data into a two-dimensional space through dimensionality reduction. **(A)** PCA is used for dimensionality reduction and visualization. **(B)** PCoA is used for dimensionality reduction and visualization. **(C)** T-SNE is used for dimensionality reduction and visualization. **(D)** NMDS is used for dimensionality reduction and visualization. Community Analysis: **(A)** We used the species accumulation curve (SAC) to describe the real situation of the disease samples. **(B)** We used volcano plots to visualize upregulated and downregulated points. MV-CVIB: Flowchart of the proposed method. The method specifically includes three fully connected layers, a two-dimensional convolutional layer, a maximum pooling layer, a PoE module, a decoding module, and an output layer.

In this study, to explore the internal structural characteristics of the pre-processed data and the degree of cognitive difference between mCRC and non-mCRC, we used various types of dimensionality reduction analysis methods (He et al., 2023), including principal components analysis (PCA) (Jiang et al., 2022), principal co-ordinates analysis (PCoA) (Wang et al., 2016), t-distributed stochastic neighbor embedding (t-SNE) (Kostic et al., 2015), and non-metric multidimensional scaling (NMDS) (Mekadim et al., 2022). The difference analysis section in Figure 3 shows an outline of the four dimensionality reduction approaches between mCRC and non-mCRC.

For PCA, the original microbial characteristic information of samples is projected into the dimension with the maximum projection information as far as possible. The advantage of PCA is that the loss of feature information after dimension reduction is minimal. The disadvantage is that in the case of complete ignorance of the data, PCA cannot better retain data information. For PCoA, it is a non-constrained dimension reduction method, and PCoA can find the most important coordinates in the distance matrix without changing the mutual position relationship between mCRC and non-mCRC. The disadvantage is that PCoA can only roughly understand the similarity or difference between samples but cannot accurately calculate the degree of difference. For t-SNE, it is a non-linear dimension reduction method, which can preserve the local features of the dataset. The disadvantage of t-SNE is that the setting of hyperparameters is relatively strict, and an improper setting will lead to poor results. Similar to PCoA, NMDS also uses the sample similarity distance matrix for dimension reduction analysis. It is worth noting that NMDS focuses on the ordering relation of values in the distance matrix. When there are more samples, NMDS can more accurately reflect the differences among samples. The disadvantage of NMDS is that it is easy to fall into the local optimal point, and it needs to run several times with different random starts to be more likely to obtain the global optimal solution. In addition, we also performed four types of dimensionality reduction visualization analysis on three CRC datasets. Overall, similar to the case of mCRC, there is a large overlap between healthy and diseased sample points. Specific experimental results are included in Supplementary material.

Considering the small number of samples and more feature information in mCRC and non-mCRC, we used four different types of dimensionality reduction methods to mine the distribution rules of samples in the dimensional space. According to the results, the spatial distribution of mCRC and non-mCRC is different, but some samples overlap in space. However, compared with non-mCRC, mCRC is formed by further deterioration on the basis of non-mCRC, so there are differences in microbial abundance features. In addition, to further show the significance index of the difference between the groups, we performed an analysis of similarities (ANOSIM) (Buttigieg and Ramette, 2014) on the mCRC dataset and three CRC datasets, respectively. As a non-parametric test method, ANOSIM has been widely used to evaluate the overall similarity and similar significance of two sets of experimental data. The results are detailed in Supplementary material. From the experimental results, there are statistically significant differences between the groups of the mCRC, CRC1, and CRC2 datasets (*R*-value of >0 and *P*-value of <0.05). There is a difference between the groups in the CRC3 dataset but not significant (*R*-value of >0 and *P*-value of >0.05), which may be related to the fact that the samples included in the dataset are early CRC patients and late CRC patients.

### 2.4. Microbial Community Analysis

To measure the species richness status in the community and judge whether the number of samples is sufficient, we used the species accumulation curve (SAC) (Gotelli and Colwell, 2001) to describe the real situation of the disease samples. With the help of the SAC, we can not only estimate the diversity difference between different communities reflected by the number of samples but also estimate the upper limit of community diversity under the condition that the number of samples is sufficient. In addition, based on the principle of statistical testing, we use the volcano plot to show the distribution of abundance level differences between the samples. The detailed results are shown in the Community Analysis section of Figure 3.

From the SAC, we can observe that the curve eventually flattens out, which confirms that the number of samples is reasonable. In other words, the number of mCRC and non-mCRC in the dataset can effectively reflect the species diversity and species richness of the samples. From the volcano plot, there are some significant points, including 24 upregulated points and 63 downregulated points, most of which have no significant difference.

### 2.5. The multi-view convolutional variational information bottleneck

We set *Y* to be a random variable. *X*^1^, *X*^2^, …, *X*^*V*^ represent a set of multi-view input random variables, and *Y* is their ground-truth labels. To make the notation more compact, a collection of data views is represented as a data point *X* = {*X*^*i*^ | *i*^*th*^ *view present*}. We set *U* to be a stochastic encoding of *X*, defined by the parameter encoder *p*(*u*|*x*; *θ*), which comes from the deep neural network of the intermediate layers in the upstream part of the model. Furthermore, in the rest of this study, *X*, *Y*, and *U* are represented as random variables, and *x*, *y*, and *u* are their multidimensional instances, respectively. *θ* is a parameter vector, and *θ* is a function parameterized by *θ*. *S* is a set.

Referring to the information bottleneck theory (Tishby et al., 2000), the purpose is to learn to encode *U*, so as to maximize the information provided to *Y* and maximize the compression to *X*. Therefore, maximizing the mutual information *I*(*U, Y*; *θ*) between *U* and *Y* can be written as follows:


(1) $$\begin{array}{l} I\left(U,Y;\theta \right)=\int p\left(u,y|\theta \right)\log\frac{p\left(u,y|\theta \right)}{p\left(u|\theta \right)p\left(y|\theta \right)}dydu. \end{array}$$

Let $I\left(U,Y;\theta \right)=\int p\left(u,y|\theta \right)\log\frac{p\left(u,y|\theta \right)}{p\left(u|\theta \right)p\left(y|\theta \right)}dydu.$ be a valid solution to maximize (1). However, given the constraint that maximizing compression imposes on *U*, we need to forget as much information about *X* as possible. Therefore, the objective function can be written as follows:


(2) $$\begin{array}{l} \underset{\theta }{\max}R_{IB}\left(\theta \right)=I\left(U,Y;\theta \right)-\beta I\left(U,X;\theta \right), \end{array}$$

where $\underset{\theta }{\max}R_{IB}\left(\theta \right)=I\left(U,Y;\theta \right)-\beta I\left(U,X;\theta \right),$ is the Lagrange multiplier greater than or equal to 0 and controls the trade-off. *I*(*U, Y*; *θ*) can make *U* to predict *Y*, and β*I*(*U, X*; *θ*) is a constraint that *U* is the minimal sufficient statistic for *X*.

We refer to the solution process by Alemi et al. (2016) for the bottleneck of deep variational information. Equation (2) can be rewritten as follows:


(3) $$\begin{array}{l} J_{DeepVIB}=\frac{1}{N}\sum_{n=1}^{N}\underset{\varepsilon \sim p\left(\varepsilon \right)}{E}\left[-\log q\left(y_{n}|f\left(x_{n},\varepsilon \right)\right)\right]\\ \;+\beta KL\left[p\left(U|x_{n}\right),r\left(U\right)\right], \end{array}$$

where ε ~ *N*(0, *I*) is denoted as the auxiliary Gaussian noise variable, and *KL* is the Kullback–Leibler divergence. It is worth noting that *f* is originally an encoding function, but in this study, it is a neural network. The introduction of *f* has a re-parameterization trick (Kingma and Welling, 2013), that is, *p*(*u*|*x*; *θ*)*dx* = *p*(ε)*dε*, where *u* = *f*(*x*, ε) can be regarded as a deterministic variable, in particular, considering that this formula can make the noise variable independent. Thus, backpropagation is used to optimize the gradient of the objective function of equation (3). Overall, the calculation will be easier. Furthermore, a multivariate Gaussian distribution with a diagonal covariance structure *u* = *f*(*x*, ε) is the target of the variational approximation posterior, and *u* = μ + σε is re-parameterized.

Since our model has multi-view input, we take into account the nearest neighbor information between each sample. Therefore, we can further improve the objective function of Deep VIB in equation (3), and*X* as a multi-view random variable can be expressed as *X*. The *p*(*U*|*x*) of equation (3) can be expressed as *p*(*U*|*x*^1^, *x*^2^, …, *x*^*V*^), with the joint of *V* available data views as the condition. We refer to the method in multimodal variational autoencoder (MVAE) (Wu and Goodman, 2018), where conditional independence between different modes of *U* and approximate *p*(*U*|*x*^*i*^) with *q*(*U*|*x*^*i*^) = *q*^~^(*U*|*x*^*i*^)*p*(*U*) is assumed. $\overset{\sim }{q}\left(U|x^{i}\right)$ is a random encoder for the i-th data view, and *p*(*U*) is a prior. Therefore, the product of multiple single-view posteriors can be considered equivalent to the joint posterior, which can be written as follows:


(4) $$\begin{array}{l} p\left(U|x^{1},x^{2},\ldots ,x^{V}\right)\propto \frac{\prod_{i=1}^{V}p\left(U|x^{i}\right)}{\prod_{i=1}^{V-1}p\left(U\right)}\\ \approx \frac{\prod_{i=1}^{V}\left[q\left(U|x^{i}\right)=q^{\sim }\left(U|x^{i}\right)p\left(U\right)\right]}{\prod_{i=1}^{V-1}p\left(U\right)}=p\left(U\right)\overset{V}{\underset{i=1}{\prod }}q^{\sim }\left(U|x^{i}\right). \end{array}$$

Equation (4) can be considered the product of experts (PoE). Considering that the product of Gaussian experts is itself a Gaussian distribution (Cao and Fleet, 2014), once the probability distribution is Gaussian, then PoE has a simple solution. Therefore, the objective formulation of the multi-view-based convolutional variational information bottleneck can be written as follows:


(5) $$\begin{array}{l} J_{MV-CVIB}=\frac{1}{N}\sum_{n=1}^{N}\underset{\varepsilon \sim p\left(\varepsilon \right)}{\text{E}}\left[-\log q\left(y_{n}|f\left(x_{n}^{1},x_{n}^{2},\ldots ,x_{n}^{V},\varepsilon \right)\right)\right]\\ \;+\beta KL\left[p\left(U\right)\prod_{i=1}^{V}\tilde{\;q}\left(U|x_{n}^{i}\right),r\left(U\right)\right]. \end{array}$$

### 2.6. Model implementation details

In MV-CVIB, we mainly input two data views, one is the microbial relative abundance matrix and the other is the nearest neighbor information, for each sample generated based on the microbial relative abundance matrix. The sample nearest neighbor information matrix can be written as follows:


(6) $$\begin{array}{l} NN_{Sample}\left(a,b\right)=\sqrt{{\sum RA\left(a_{i}-b_{i}\right)}^{2}}, \end{array}$$

where *NN*_*Sample*_ represents the sample nearest neighbor information matrix, *NN*_*Sample*_ represents the relative abundance matrix, and *a*_*i*_ and *b*_*i*_ represent the i-th element of the row vector and column vector, respectively.

To avoid overfitting, dropout and early stopping are applied in this study. Dropout greatly reduces the size of the neural network, allowing the neural network to learn local features in the data. Early stopping can stop training early when overfitting occurs. We used the dedicated stochastic encoder *b*_*i*_ to embed different views of gut microbiome data. *f*_*mlp*_ represents a multilayer perceptron (MLP). For the data of both the above views, we used the SiLU (Hendrycks and Gimpel, 2016) activation function for fully connected layers and used dropout (*p* = 0.2) during training.

We used a logistic regression model *q*(*y*|*u*) = σ(*f*_*d*_(*u*)) with a logistic sigmoid function in the decoder and $f_{d}\left(u\right)=w^{T}u+b$. The purpose is to perform binary classification operations. *y* models two diagnostic CRC labels, such as mCRC and non-mCRC. Furthermore, for other published CRC datasets in this study, *y* models two diagnosed disease labels, such as CRC or healthy. In addition, in equation (5) mentioned above, *r*(*U*) and *r*(*U*) are spherical Gaussian distributions with *K* dimensions, where *r*(*U*) = *p*(*U*) = *N*(0, *I*). We set *K* = 256 and β = 10^−5^.

All experiments were performed under Windows 10 with NVIDIA GTX 1650 GPU and CUDA 10.2 installed, where the machine's processor is AMD Ryzen7 4800H. The source code and data are available at: https://github.com/cuizhensdws.

### 2.7. Performance evaluation

To evaluate the classification performance of the model more accurately and comprehensively, inspired by DeepMicro, we design a similar evaluation scheme. The ratio of the training set and test set in the mCRC dataset is adjusted to 8:2. It is worth noting that the random partition seed is also set the same as DeepMicro, which guarantees a fair random training-test split. Furthermore, for the published CRC dataset, we also adopted the same dataset partitioning scheme. The above settings can further reduce the information leakage and improve the efficiency of the response model more accurately.

We used a stratified 5-fold cross-validation applied to the training set and calculated the AUC score through the validation set, and the epoch with the best parameters among all epochs was selected. The AUC can be written as follows:


(7) $$\begin{array}{l} AUC=\frac{\sum_{i\in positiveclass}rank_{i}-\frac{M*\left(1+M\right)}{2}}{M*N} \end{array}$$

where *M* is the number of positive samples, and *N*is the number of negative samples.

## 3. Results

### 3.1. Predictive performance of MV-CVIB on mcrc datasets

To evaluate the performance of MV-CVIB, we compared the existing advanced methods, including MVIB (Grazioli et al., 2022), PopPhy-CNN (Reiman et al., 2020), DeepMicro (Oh and Zhang, 2020), random forest (RF), multilayer perceptron (MLP), and SVM. To be fair, these methods are executed multiple times. Considering that different devices and parameter settings may affect the prediction results, we can only ensure that each method is relatively optimal rather than absolutely optimal. It is important to note that DeepMicro is a framework consisting mainly of dimension reduction modules and classification modules. The DeepMicro model does not specify which combination has the best predictive performance. Therefore, we compared all the method combinations.

**Figure 4:**
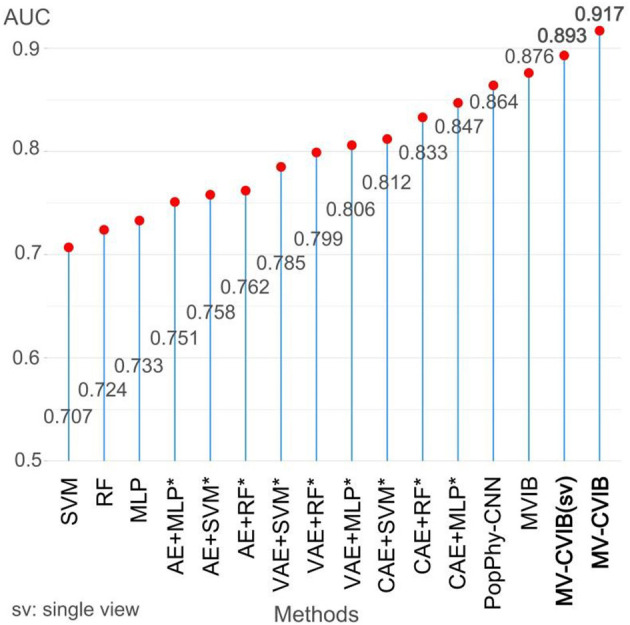
Comparison of AUC values of our method with state-of-the-art methods on the mCRC dataset. The methods with asterisks are all from the DeepMicro model, and these methods are a combination of methods in DeepMicro. The best performing AUC is indicated in black bold.

Our method consists of two parts, one is MV-CVIB which contains the nearest neighbor information, and the other is MV-CVIB (single view) which uses only microbiome abundance data. For PopPhy-CNN, we modified the source code so that it is roughly consistent with our model framework. The original PopPhy-CNN provided different experimental procedures. To ensure the consistency of the verification test, we made corresponding framework adjustments. For SVM and RF, we used the same grid search to set the hyperparameters, referring to MetAML (Pasolli et al., 2016). Figure 4 shows the AUC values for each method. Specifically, the AUC value of MV-CVIB reached 0.917, better than 0.893 of MV-CVIB (single view). In other words, the nearest neighbor information helped improve the prediction performance of MV-CVIB by 2.4%. Moreover, the AUC value of MV-CVIB and MV-CVIB (single view) both exceeded that of MVIB, and the AUC value of MV-CVIB is 4.1% higher than that of MVIB. Slightly lower than MVIB is PopPhy-CNN, which has an AUC value of 0.864. Multiple combinations in the DeepMicro framework achieve an AUC value >0.75.

### 3.2. Predictive performance of MV-CVIB on CRC datasets in published studies

**Figure 5:**
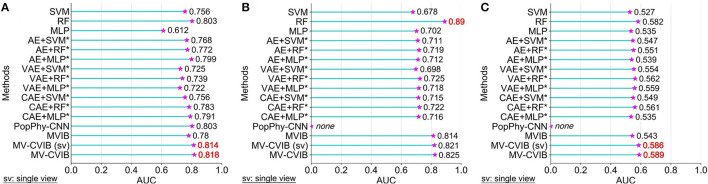
Comparison of AUC values of our method with state-of-the-art methods on three CRC datasets. The methods with asterisks are all from the DeepMicro model, and these methods are a combination of methods in DeepMicro. On the Colorectal dataset, we follow the partial AUC results from DeepMicro. Specifically including AE + SVM^*^, VAE + SVM^*^, and CAE + RF^*^, the results of other combinations are derived from this study. Since DeepMicro did not use Colorectal-EMBL and Early-Colorectal-EMBL datasets, the results on these two datasets are also derived from this study. The best performing AUC is indicated in red font. **(A)** The AUC value of each method on the Colorectal dataset. **(B)** The AUC value of each method on the Colorectal-EMBL dataset. **(C)** The AUC value of each method on the Early-Colorectal-EMBL dataset.

In this study, to more comprehensively evaluate our predictive model, we also performed predictive experiments on three CRC datasets. Figure 5 shows the AUC values for each method.

First, on the Colorectal dataset, the AUC value of the proposed model was 0.818, while the AUC value of MV-CVIB (single view) was 0.814, both of which were superior to other advanced models. Interestingly, RF and PopPhy-CNN both have the same AUC of 0.803. The AUC of MVIB is only 0.78, and the performance of the model is mediocre. In DeepMicro, the AUC value of AE + MLP was 0.799. The predictive effect of CAE+MLP was slightly lower than that of AE + MLP, and the AUC was 0.791.

Second, on the Colorectal-EMBL dataset, as shown in Figure 5, the AUC value for RF is 0.89, which is higher than any other method. This was followed by MV-CVIB and MV-CVIB (single view) with AUC values of 0.825 and 0.821, respectively. Compared with MVIB, the prediction performance of MV-CVIB is improved by 1.1%. Compared with RF, the prediction performance of MV-CVIB is reduced by 6.5% and that of MVIB is reduced by 7.6%. It is worth noting that we cannot get the predicted results of PopPhy-CNN because there is an infinite loop in the experiment process.

Finally, on the Early-Colorectal-EMBL dataset, as shown in Figure 5, none of the methods has an AUC value above 0.6. According to the previous ANOSIM analysis, there are differences between the groups on the Early-Colorectal-EMBL dataset but not significant. Therefore, we speculate that this may be a reason for the AUC of each method to be less than 0.6. Compared with other advanced methods, MV-CVIB achieved an AUC value of 0.589, while MV-CVIB (single view) was slightly lower, with an AUC value of 0.586. Compared with MVIB, MV-CVIB improved performance by 4.6%. Once again, RF (AUC = 0.582) showed excellent performance on this dataset, outperforming all methods except ours. Moreover, in DeepMicro, any autoencoder combined with RF achieved a high AUC value. As on the Colorectal-EMBL dataset, PopPhy-CNN still produces an infinite loop on the Early-Colorectal-EMBL dataset.

### 3.3. Ablation experiments

**Figure 6:**
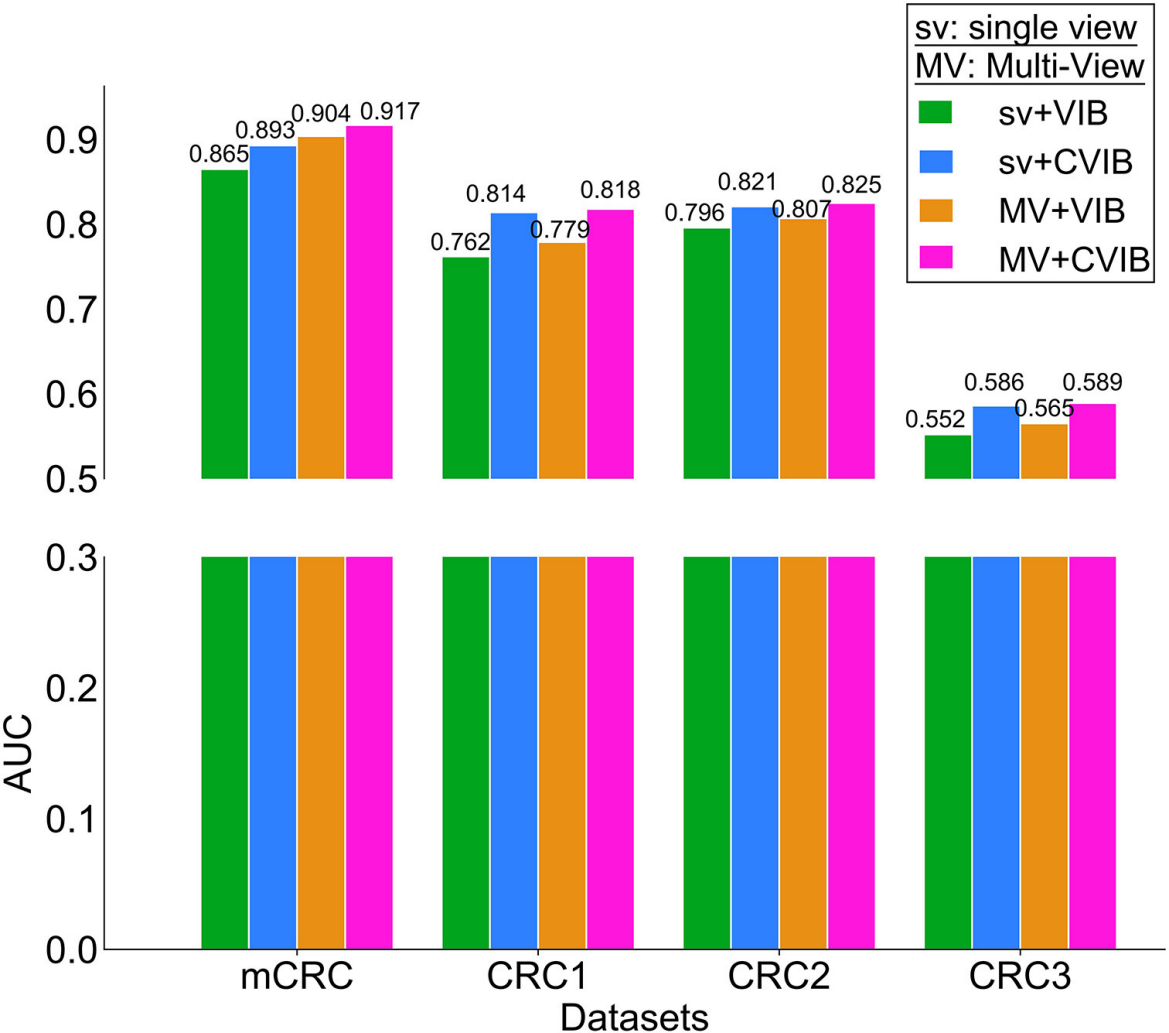
AUC comparison of different combinations of the proposed method on different datasets.

Considering that we have introduced multi-view, convolution, and pooling modules in MV-CVIB, to verify the impact of this module on the overall performance of the method, we set up multiple ablation experiments on the mCRC dataset and three CRC datasets. Figure 6 shows the AUC values of different combinations of the proposed method on different datasets. From the results of the ablation experiments, it can be observed that on the mCRC dataset, the impact of multi-view on the performance of the method is slightly higher than that of the convolution module. However, on the three CRC datasets, the impact of the convolution module on the performance of the method is slightly higher than that of the multi-view.

Overall, according to the results of the ablation experiments, we speculate that when the number of samples is small, multi-view may be easier to take advantage of the prediction performance; when the number of samples is large, the convolution module may be more likely to take advantage of the prediction performance.

### 3.4. The gut microbial diversity is different in mcrc and non-mcrc patients

**Figure 7:**
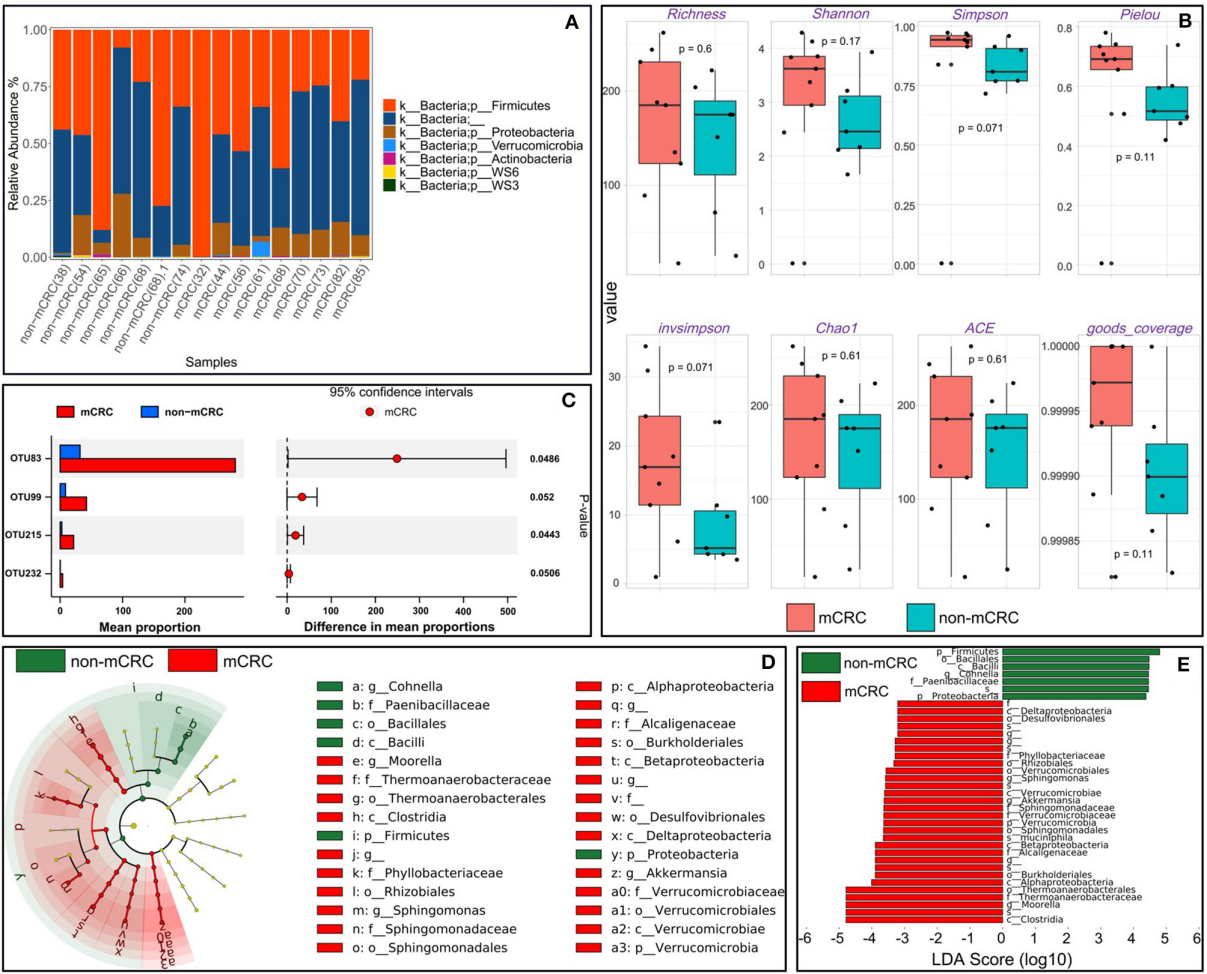
**(A)** Microbiota (phylum level) stacked bar graphs of the non-mCRC group and mCRC group. **(B)** To better describe the microbial richness and uniformity of the intestinal tract, we used the alpha diversity index to measure the intestinal ecosystem from different perspectives. **(C)** To further mine the differences between non-mCRC and mCRC samples, we used STAMP to output significantly different OTUs within the 95% confidence interval. **(D)** LDA effect size (LEfSe) analysis was used to discover and interpret biomarkers that were statistically different between non-mCRC and mCRC patients. **(E)** Histogram of the distribution of LDA values.

The stacked bar graphs of the microbiota (phylum level) of the non-mCRC group and the mCRC group are shown in Figure 7A. In their gut, the microbial community was dominated by Firmicutes and Proteobacteria. Among them, a small number of Verrucomicrobia were present in a 61-year-old mCRC patient. It can be observed from the figure that as the age of mCRC patients increases, the relative abundance of Firmicutes tends to decrease overall.

As shown in Figure 7B, to better describe the microbial richness and uniformity of the intestinal tract, we used the alpha diversity index to measure the intestinal ecosystem from different perspectives (Wang et al., 2018). It specifically includes eight indicators: richness, Shannon, Simpson, Pielou, invsimpson, Chao1, ACE, and goods coverage. Taken together, compared with non-mCRC patients, the number and diversity of intestinal communities in mCRC patients tended to increase, and the evenness index of intestinal communities in mCRC patients was significantly increased. In addition, from the perspective of goods coverage, the indices of non-mCRC and mCRC samples are close to 1, which indicates that the sequencing depth is reasonable, that is, the depth has basically covered all species in the sample. We also performed alpha diversity analysis on the three CRC datasets, and the specific analysis results are included in Supplementary material.

### 3.5. Potential biomarker identification with statistical differences

To further mine the differences between non-mCRC and mCRC samples, we used STAMP to output significantly different OTUs within the 95% confidence interval (Parks et al., 2014). As shown in Figure 7C, the mean proportion value at OTU83 was significantly higher in mCRC patients than in non-mCRC patients. Second, at OTU99, OTU215, and OTU232, mCRC patients were also higher than non-mCRC patients. Interestingly, the taxonomy of OTU215 was accurate to the species, specifically *Propionibacterium acnes*. Not only that, we also used the LDA effect size (LEfSe) (Segata et al., 2011) analysis to discover and explain the biomarkers with statistical differences between non-mCRC and mCRC patients. As shown in the clade diagram of Figure 7D, yellow indicates species without significant differences, and both red and green indicate significant differences. Among them, green nodes represent microbial groups that play an important role in non-mCRC samples, and red nodes represent microbial groups that play an important role in mCRC samples. In the histogram of LDA value distribution in Figure 7E, we can clearly find that there are far more biomarkers with statistical differences in mCRC samples than in non-mCRC samples. Therefore, this may be more conducive to the prediction of metastatic disease in CRC patients, so as to make early diagnosis and treatment.

### 3.6. Patients' age and metastatic risk assessment

As mentioned earlier in this study, with the development of a standardized multidisciplinary team consultation model, the survival rate of non-mCRC patients has been significantly improved. However, the metastatic nature of non-mCRC cannot be ignored, and the therapeutic effect of most chemotherapy drugs on mCRC is limited. Therefore, compared with non-mCRC, the survival rate of mCRC is extremely low. To assess the relationship between patient age and metastatic risk of non-mCRC, we constructed a risk model to obtain a risk score. Patients will be divided into high-risk and low-risk groups based on risk scores. Ultimately, we explored the relationship between microbiota expression and patient survival.

**Figure 8:**
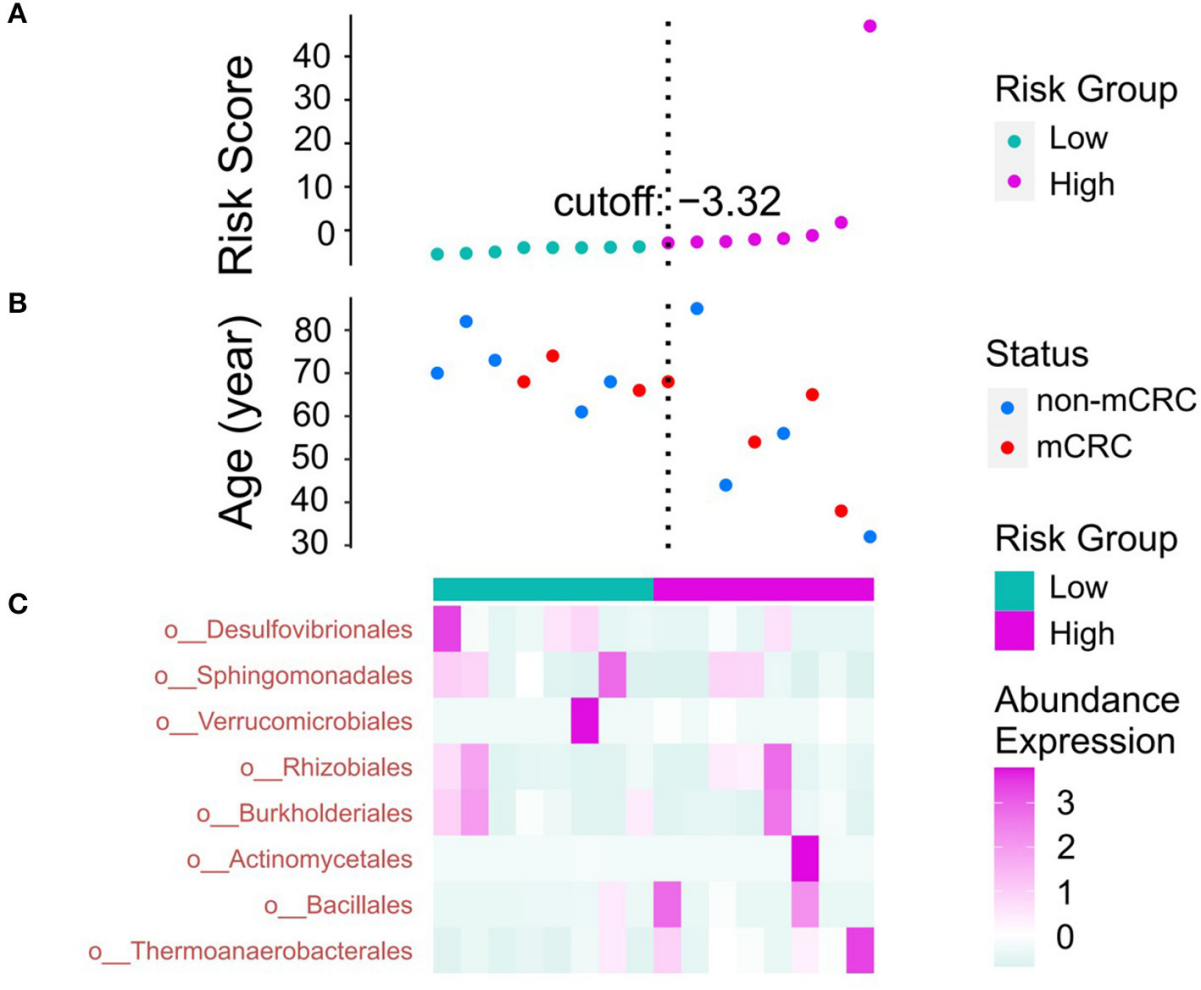
**(A)** According to the risk value, the high risk group and the low risk group are divided. **(B)** Scatter plot of the relationship between patient age and risk status. **(C)** The heat map of the abundance expression of the bacterial group (order level).

As shown in Figure 8, patients with risk scores were divided into high-risk and low-risk groups according to the cutoff value. Combining Figures 8A, B, it can be observed that as the risk score increases, the age of patients presents a downward trend, and the age span of the high-risk group is larger than that of the low-risk group. There is a possibility of cancer metastasis in all segments. It can be observed from Figure 8C that the expression of Desulfovibrionales showed a trend from high to low from left to right, while Thermoanaerobacterales and Actinomycetales showed a trend of gradually increasing expression. From Figures 8A, C, it can be observed that Thermoanaerobacterales and Actinomycetales are positively correlated with risk scores, and Desulfovibrionales are negatively correlated with risk scores. Other flora showed irregular expression trends. Desulfovibrionales produce hydrogen sulfide, a genotoxic compound in the gut. This substance can destabilize the genome or chromosomes (Dahmus et al., 2018; Zhao et al., 2022). Several recent studies have shown that genomic instability is found in more than 80% of sporadic CRCs. Actinomycetales are an important gut flora. Actinomycetales involved in CRC development have different characteristics compared with healthy microbiota (Rebersek, 2021; Li et al., 2023).

Overall, the higher the risk score, the worse the prognosis, and the higher the expression of Desulfovibrionales, the better the prognosis, which may be the beneficial flora before cancer metastasis; while the higher the expression of Thermoanaerobacterales and Actinomycetales, the worse the prognosis, which may be the bad flora after cancer metastasis.

## 4. Discussion

Many complex reasons and limitations make microbiome-based disease prediction a challenging task. It is mainly reflected as follows: (1) The composition of the human microbial community is very complex, and the boundaries between the bacterial communities are fuzzy. (2) There are various ways to generate microbial community characteristic data, which leads to data heterogeneity. (3) Human health status is dynamic rather than fixed, and healthy samples are not absolute, which may increase data noise and outliers. (4) Conventional microbiome-based disease prediction mostly adopts the health-disease classification method, ignoring the deterioration process of the disease. We adopted a new classification schema: disease-disease instead of health-disease. We focussed on identifying more severe diseases, especially cancer and cancer metastases, from disease samples. This facilitates exploration and reveals the underlying properties of disease exacerbation. It is meaningful for non-mCRC patients. We can obtain potential biomarkers through the analysis of differences in the bacterial flora of patients and explore the biological process and development rules of CRC metastasis on the basis of the microbiome. This is conducive to further expanding the treatment options for non-mCRC patients and improving the prognosis of non-mCRC patients.

We employed a variety of microbiome analysis methods to explore the diversity of non-mCRC and mCRC. From the experimental results, the communities of non-mCRC and mCRC were quite different, the distribution of the flora was complex and diverse, and the flora composition of different samples was different. Compared with the state-of-the-art methods for microbiome-based disease prediction, the proposed method MV-CVIB achieved higher AUC values on the mCRC dataset. In addition, in order to more comprehensively evaluate MV-CVIB and verify its generalization ability, we collected datasets from three published studies and conducted experiments. The number of samples in the three public CRC datasets does not exceed 200, which belongs to high-dimensional small sample data. This limitation may affect the experimental results. Therefore, in future, we can use transfer learning or data augmentation. The experimental results show that MV-CVIB achieves higher AUC values on two of the three datasets. Based on the predicted results, we performed a statistical analysis of potential biomarkers in non-mCRC and mCRC. Finally, we modeled the risk score, explored the age trend of the risk score, and screened out those bacterial orders that had positive and negative effects on patients.

## 5. Conclusion

In this study, we propose a deep learning approach based on a multi-view convolutional variational information bottleneck for the prediction of mCRC. The multi-view contains species abundance data and sample field information, where the sample neighborhood information is obtained based on the species abundance data, which ensures that our method will not introduce additional noise when inputting multi-view data, and has a better time and space complexity. Our results demonstrate that the method has good predictive performance. However, on the Colorectal-EMBL dataset, all deep learning methods are not as effective as RF, which may be related to the internal structural characteristics of the dataset. We explored the degree of difference between non-mCRC and mCRC from various perspectives, analyzed those statistically significant differences in flora, and constructed an age risk assessment model to explore the rules of age and cancer metastasis. Of course, there are also some deficiencies in this study, mainly two points. First, due to concerns about patient privacy and medical ethics, the number of samples obtained is small, which may cause the model to fall into overfitting to small samples. Second, the prediction model is only for CRC data and does not consider other disease data, which may lead to a lack of generalizability of the model. We will also develop more effective methods for more complex microbiome-based disease phenotype prediction and improve the scalability of the prediction method as much as possible.

## Data availability statement

The datasets presented in this study can be found in online repositories. The names of the repository/repositories and accession number(s) can be found at: https://www.ncbi.nlm.nih.gov/, PRJNA531761.

## Author contributions

ZC proposed and designed the algorithm. YW and D-SH demonstrated the effectiveness of the method and analyzed the experimental data. Q-HZ, S-GW, and YH drafted the manuscript. All authors contributed to the article and approved the submitted version.
