# Supplementary material for: MV-CVIB: a microbiome-based multi-view convolutional variational information bottleneck for predicting metastatic colorectal cancer
Source: Front Microbiol. 2023 Aug 22;14:1238199. doi: 10.3389/fmicb.2023.1238199 (PMC10477591; doi:10.3389/fmicb.2023.1238199)
Supplement: Supplementary file 1 [file Data_Sheet_1.pdf]

*Supplementary Material*

# MV-CVIB：A Microbiome-Based Multi-View Convolutional Variational Information Bottleneck for Predicting Metastatic Colorectal Cancer

**Zhen Cui[1], Yan Wu[2*], Qin-Hu Zhang[3], Si-Guo Wang[1], Ying He[1], De-Shuang Huang[3*]**

***Correspondence:** Yan Wu: yanwu@tongji.edu.cn; De-Shuang Huang: dshuang@eias.ac.cn

## 1 Supplementary Figures

### 1.1 Supplementary Figure

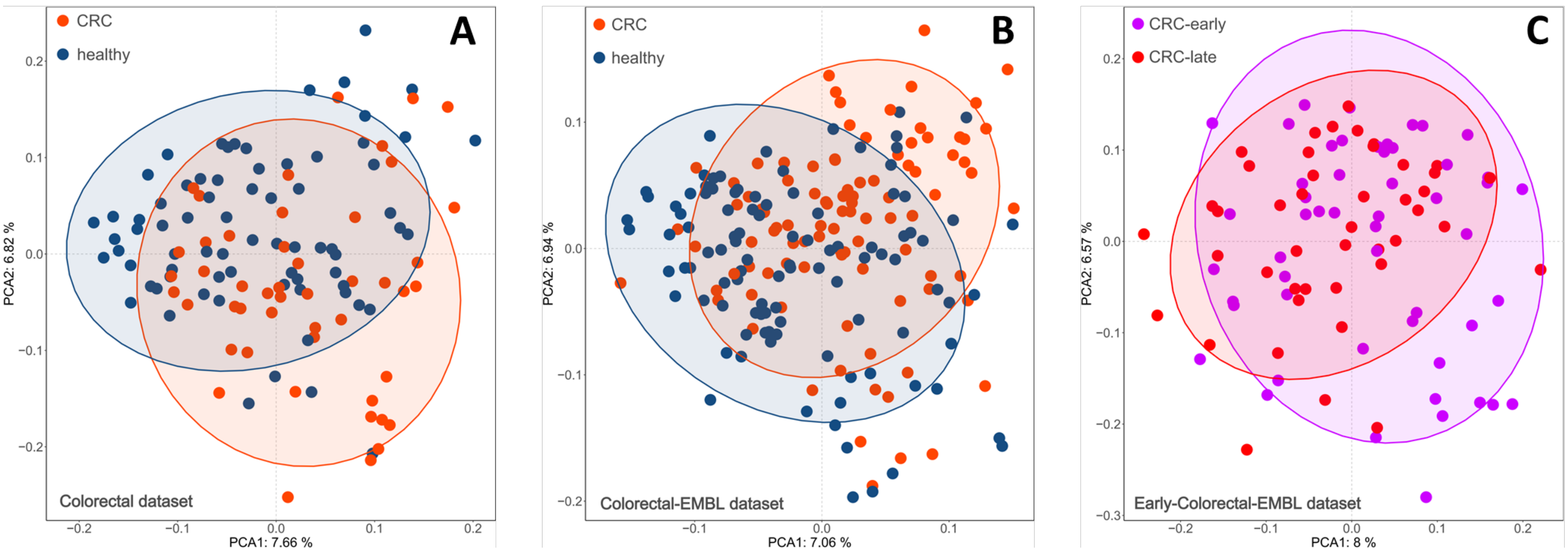


**Supplementary Figure 1.** PCA visualization on three CRC datasets.

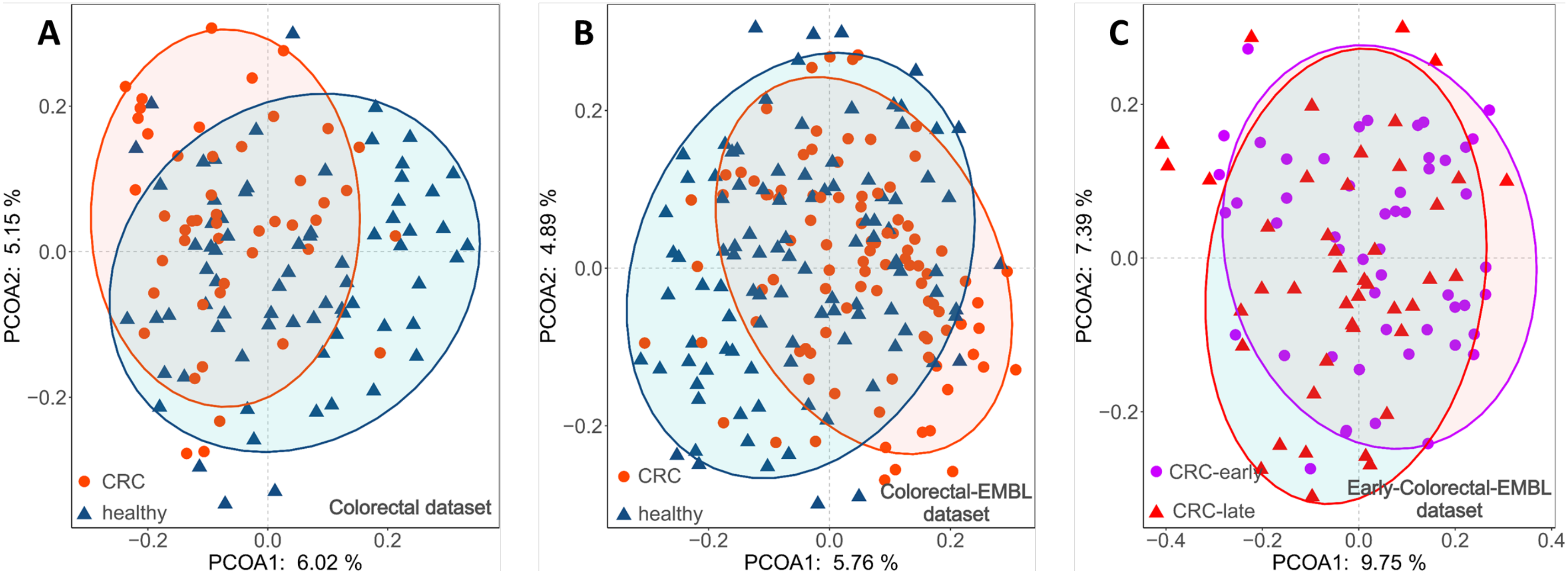


**Supplementary Figure 2.** PCoA visualization on three CRC datasets.

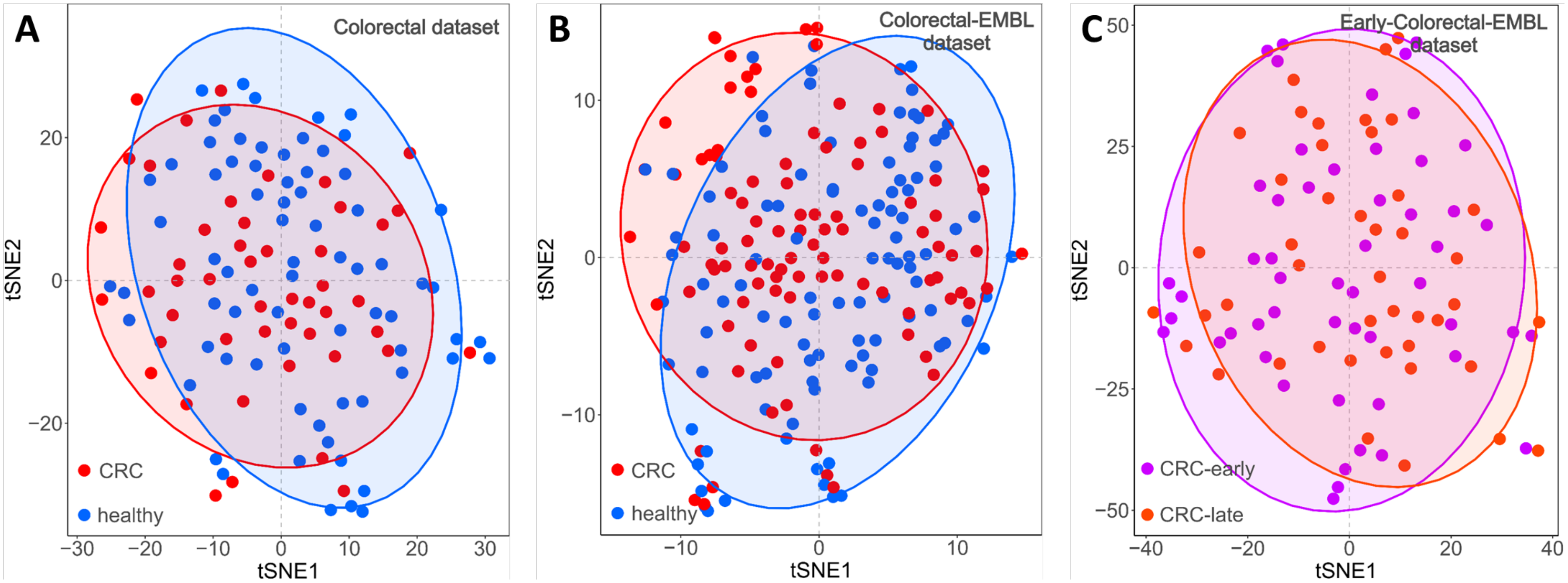


**Supplementary Figure 3.** T-SNE visualization on three CRC datasets.

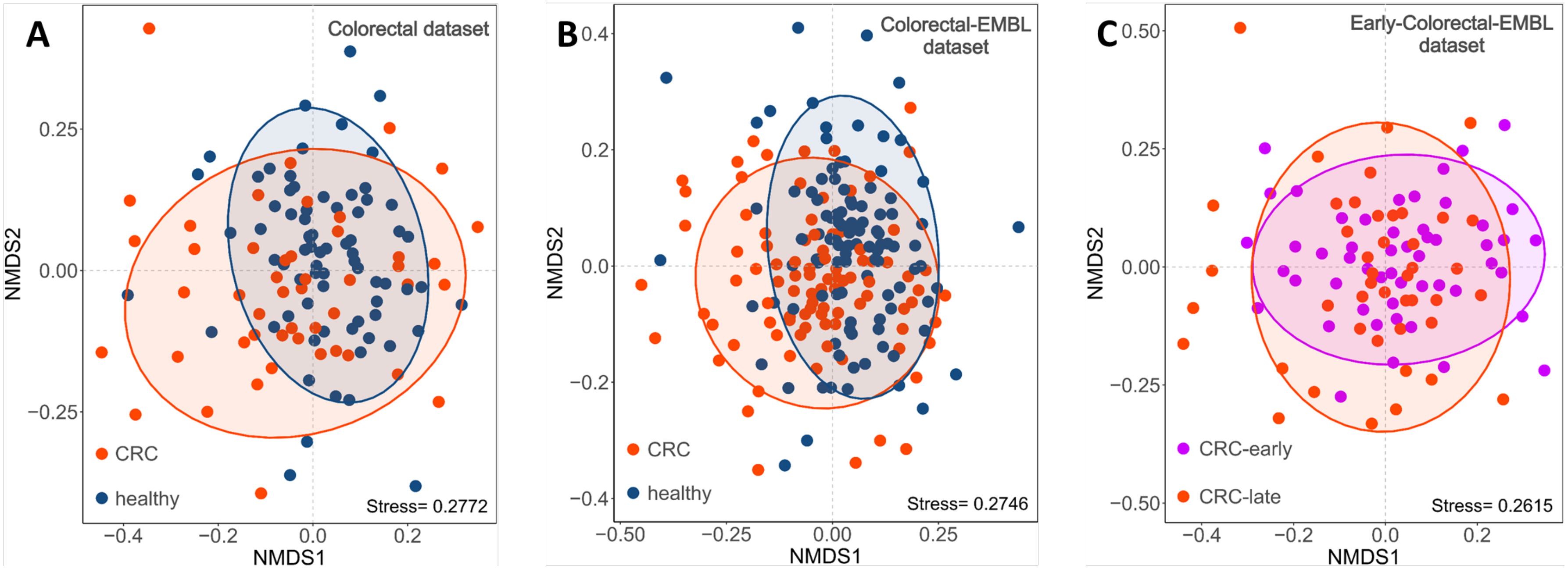


**Supplementary Figure 4.** NMDS visualization on three CRC datasets.

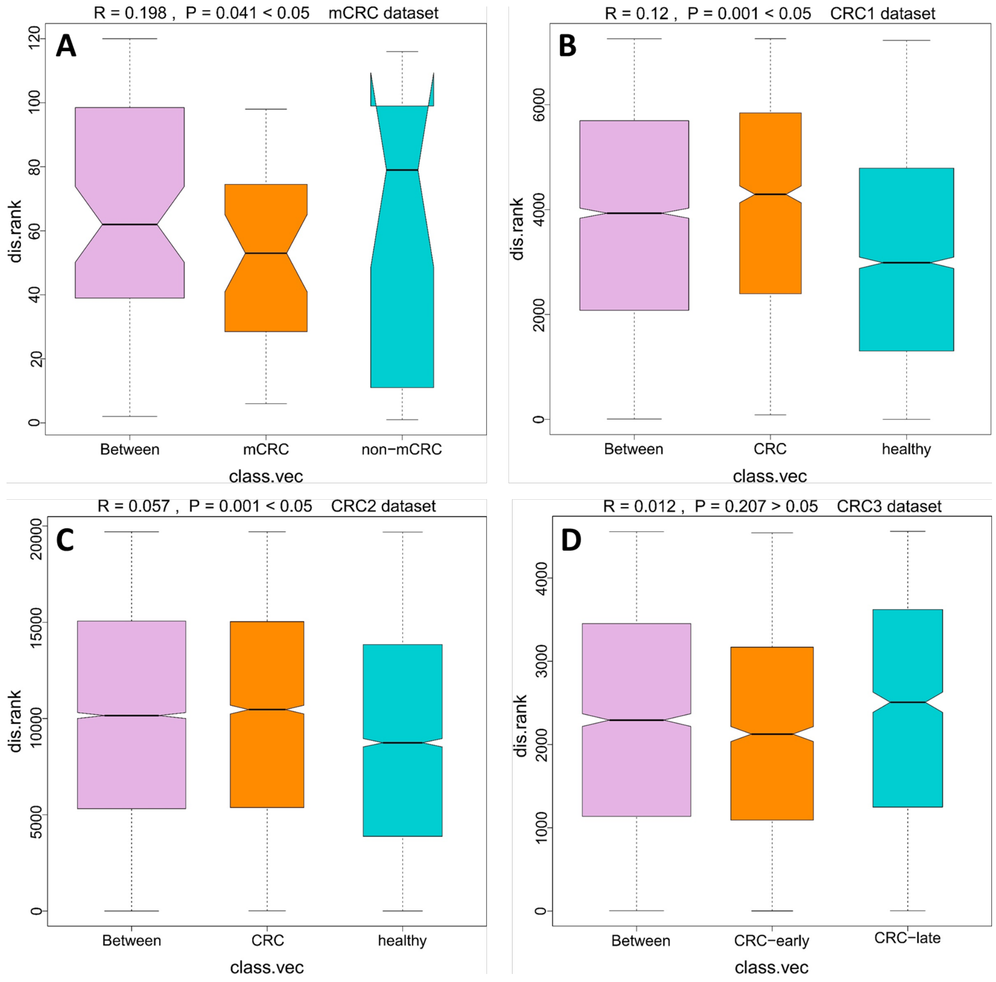


**Supplementary Figure 5.** ANOSIM analysis results on the mCRC dataset and three CRC datasets. As a non-parametric test method, ANOSIM has been widely used to evaluate the overall similarity and similar significance of two sets of experimental data. The method mainly includes two numerical results: one is the R-value, which is used to indicate whether there is a difference between different groups; the other is the P-value, which is used to indicate whether there is a statistically significant difference. From the experimental results, there are statistically significant differences between the groups of the mCRC, CRC1 and CRC2 datasets (R-value>0, P-value<0.05). There is a difference between groups in the CRC3 dataset but not significant (R-value>0, P-value>0.05), which may be related to the fact that the samples included in the dataset are early CRC patients and late CRC patients.

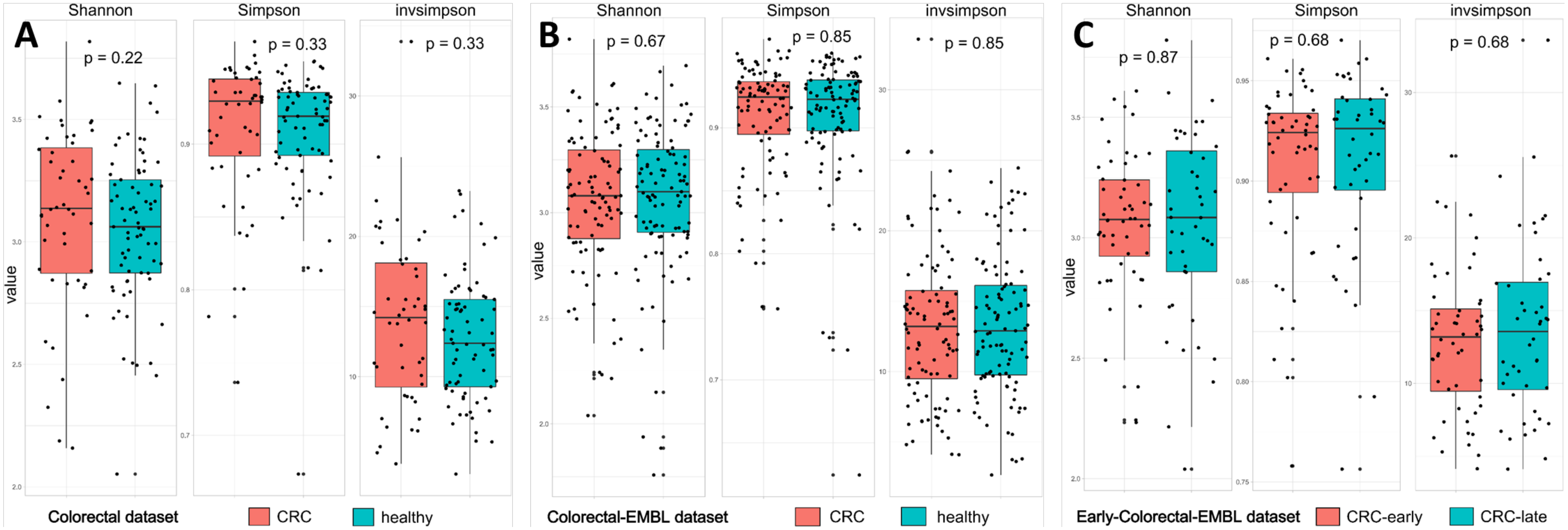


**Supplementary Figure 6.** Alpha diversity analysis on three CRC datasets.
